# Chemotherapy induced abnormal aortic function assessed by magnetic resonance imaging

**DOI:** 10.1186/1532-429X-11-S1-O55

**Published:** 2009-01-28

**Authors:** Narumol Chaosuwannakit, Ralph D'Agostino, Craig A Hamilton, Julia Lawrence, Frank M Torti, William C Little, W Gregory Hundley

**Affiliations:** Wake Forest University School of Medicine, Winston-salem, NC USA

**Keywords:** Trastuzumab, Cardiovascular Magnetic Resonance, Arterial Stiffness, Pulse Wave Velocity, Herceptin

## Introduction

Abnormally increased cardiovascular stiffness is an independent predictor of cardiovascular events. Compared to age and gender matched healthy individuals, cancer survivors previously exposed to chemotherapy experience an elevated risk of cardiac events. We hypothesized that the administration of chemotherapy may increase arterial stiffness above that observed in age and gender matched controls.

## Purpose

To determine if chemotherapy increases arterial stiffness within the thoracic aorta.

## Methods

We performed a prospective, case-control study of 23 participants that received chemotherapy (cases) with 13 that did not (controls). For a variety of malignant neoplasms, 22 participants received anthracyclines and/or other therapies including trastuzumab or Herceptin (n = 5), paclitaxel (n = 7) or cyclophosphamide (n = 11). Each participant underwent phase-contrast cardiovascular magnetic resonance imaging (PC-CMR) at baseline (before chemotherapy administration in cases) and 3 to 4 months later. During CMR, thoracic aortic distensibility and pulse wave velocity (PWV) were determined according to the following formula:

Aortic distensibility (10^-3^ mmHg^-1^) = [maximal aortic area - minimal aortic area]/[pulse pressure × minimal aortic area]

PWV (m/s) = Distance between ascending and descending thoracic aorta/Transit time of the flow wave

PC-CMR parameters included an 8 mm thick slice with a 192 × 108 matrix, a 36 cm FOV, a 15° flip angle, a 76.5 ms TR, a 3.14 ms TE and a through-plane velocity encoding of 150 cm/sec. A nonferromagnetic brachial blood pressure cuff was applied to record heart rate and blood pressure noninvasively during the phase-contrast acquisition. To compare groups at their follow-up visit, four one-way analysis of covariance models (ANCOVA) were fit where factors known to influence aortic stiffness were included as covariates in the model.

## Results

**Figure 1:**
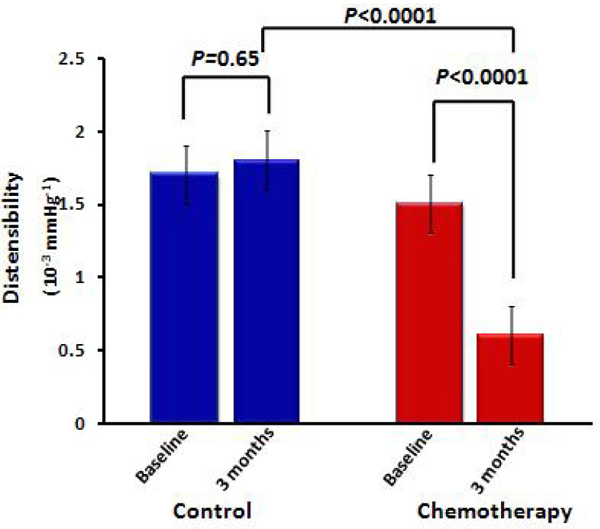
Figure 1

**Figure 2:**
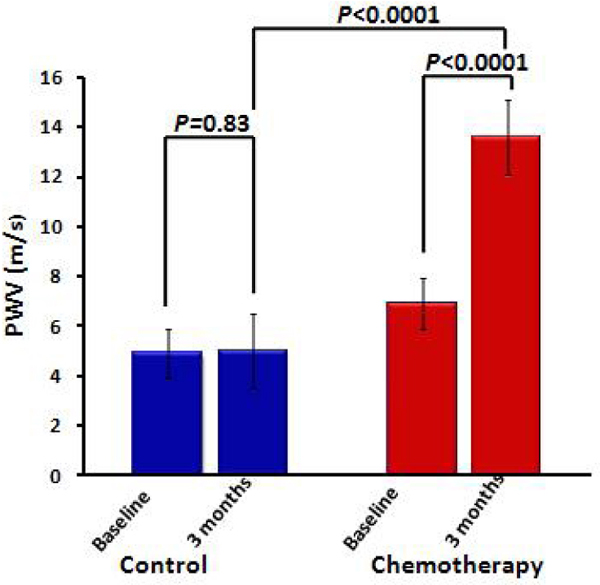
Figure 2

At 3 months, arterial stiffness (distensibility and PWV) remained similar in the control participants. However, in the participants receiving chemotherapy, aortic stiffness markedly increased as evidenced by a decrease in distensibility and an increase in PWV. When we compared the participants receiving chemotherapy with controls directly using an ANCOVA model that adjusted for baseline aortic stiffness, age, gender, body mass index, systolic blood pressure, heart rate, pulse pressure, medication use, and the presence of hypertension, diabetes and hyperlipidemia, distensibility and PWV were significantly different between controls and chemotherapy recipients (p < 0.0001; Figures 1 and 2).

## Conclusion

Patients receiving relatively short courses of chemotherapy (3 months) experience a significant increase in vascular stiffness (manifest as both reduced aortic distensibility and increased PWV) compared to healthy controls. These results indicate that previously regarded cardiotoxic chemotherapy adversely increases cardiovascular stiffness, a known independent predictor of cardiovascular events.

